# Development of blood biomarker for Alzheimer disease using neuron‐derived extracellular vesicles

**DOI:** 10.1002/alz.088316

**Published:** 2025-01-09

**Authors:** Takashi Kudo, Shoshin Akamine

**Affiliations:** ^1^ Iseikai International General Hospital, Osaka Japan; ^2^ Osaka University Graduate School of Medicine, Toyonaka Japan

## Abstract

**Background:**

We have developed a technology for isolating extracellular vesicles (EVs) released from the central nervous system present in plasma.

**Method:**

Initially, we differentiated induced pluripotent stem cells (iPS) into neurons to examine the membrane surface molecules of neuron‐derived EVs in culture media. Our analysis revealed a specific interest in neuron‐specific APLP1. Subsequently, when we fractionated plasma using an iodixanol density gradient, we identified CD63, CD9, and Syntenin1 in the fraction containing APLP1. This confirmed the presence of APLP1‐positive EVs in plasma. Our protocol for isolating nerve‐derived EVs involves first separating total EVs from plasma using size exclusion chromatography, followed by isolating nerve‐derived EVs through immunoprecipitation with an anti‐APLP1 antibody. Using this method, we successfully isolated nerve‐derived EVs from plasma obtained from Alzheimer's disease patients and analyzed their protein content using digital ELISA.

**Result:**

Tau in EVs measured by digital ELISA was significantly correlated with Alzheimer's disease biomarkers in cerebrospinal fluid.

**Conclusion:**

Neuron‐derived EVs obtained through our developed method could serve as potential biomarkers for Alzheimer disease.

